# Isorhamnetin 3-*O*-robinobioside from *Nitraria retusa* leaves enhance antioxidant and antigenotoxic activity in human chronic myelogenous leukemia cell line K562

**DOI:** 10.1186/1472-6882-12-135

**Published:** 2012-08-22

**Authors:** Jihed Boubaker, Mohammed Ben Sghaier, Ines Skandrani, Kamel Ghedira, Leila Chekir-Ghedira

**Affiliations:** 1 Laboratory of Cellular and Molecular Biology, Faculty of Dental Medicine, University of Monastir, Rue Avicenne, Monastir 5000, Tunisia; 2 Unity of Pharmacognosy/Molecular Biology, Faculty of Pharmacy, University of Monastir, Rue Avicenne, Monastir 5000, Tunisia

## Abstract

**Background:**

In this report, the isorhamnetin 3-*o*-robinobioside and its original extract, the ethyl acetate extract, from *Nitraria retusa* leaves, were evaluated for their ability to induce antioxidant and antigenotoxic effects in human chronic myelogenous leukemia cell line.

**Methods:**

*Nitraria retusa* products properties were carried out by firstly evaluating their effects against lipid peroxidation induced by H_2_O_2_, using the thiobarbituric acid reactive substances species (TBARS) assay, and proceeding to the assay of cellular antioxidant activity, then doing the comet assay.

**Results:**

The isorhamnetin 3-*o*-robinobioside showed a protective effect against lipid peroxidation induced by H_2_O_2_. The same natural compound and ethyl acetate extract inhibited oxidation induced by 2,2′-azobis (2-amidinopropane) dihydrochloride in human chronic myelogenous leukemia cells with respectively 50% inhibitory concentration values of 0.225 mg/ml and 0.31 mg/ml, reflecting a significant antioxidant potential. The same two products inhibited the genotoxicity induced by hydroxyl radicals in the same human cell line (by 77.77% at a concentration of 800 μg/ml and by 80.55% at a concentration of 1000 μg/ml respectively).

**Conclusions:**

The **i**sorhamnetin 3- *o*-robinobioside and its original extract, the ethyl acetate extract, from *Nitraria retusa* leaves, have a great antioxidant and antigenotoxic potential on human chronic myelogenous leukemia cell line K562.

## Background

Some flavonoids are more selective towards cancer cells and may have the potential for reducing the side-effects compared to other anticancer drugs [1]. In fact, flavonoids cause cell cycle arrest in G2/M phase, decreased cyclin B1 and cyclin-dependent kinase 1 in cancer cells in a p53 independent manner [2]. Environmental mutagens and carcinogens are instrumental in initiation, promotion, and progression of several kinds of cancers. The exposure to these xenobiotics is often unavoidable and therefore creates a great risk to human health. A complimentary approach is to render hosting organism more resistant to the attack of mutagens and carcinogens by supplementing the diet with chemopreventive agents [3]. The intake of sufficient amounts of antimutagens and/or anticarcinogens is believed to confer a preventive effect on the initiation and development of human cancers [4]. Oxidative stress is thought to be an important factor contributing to their development. Flavonoids have also been found to inhibit a wide range of enzymes involved in the oxidation systems such as 5-lipoxygenase, cyclooxygenase, monooxygenase, or xanthine oxidase [5]. These biological activities are related to their antioxidative effects [6]. Phytochemicals are secondary metabolic products produced by plants in response to the environmental stresses. Laboratory studies have demonstrated that some plants when eaten in whole or their active constituents are taken in isolation, show adequate protective effects against human carcinogenesis and mutagenesis [7]. The protective effect of phytochemicals may be ascribed to their potential to destroy the reactive oxidative species (ROS) that initiate carcinogenesis through the oxidative damage of DNA [8]. Herbal remedies and phytotherapy drugs containing active principles are currently developed to protect against electrophile (e.g. free radical) attack to DNA and its widespread outcomes such as ageing and cancers [9], this is the case for *Nitraria retusa* (Forssk.) Asch, which is a genus of Nitrariaceae family. Its fleshy red fruits are eaten by humans and are used to prepare drinks. The leaves serve as supplement for tea and are used as poultice. The ashes of this species have the ability to remove fluids of infected wounds. Fresh leaves of *Nitraria retusa* decoction is used in Morocco in case of poisoning, upset stomach, ulcers, gastritis, enteritis, heartburn, colitis and colonic abdominal pain [10].

For initial antioxidant screening of foods and dietary supplements, cell culture models provide an approach that is cost-effective, relatively fast, and address some issues of uptake, distribution, and metabolism. The objective of this research was to use a quantitative cellular antioxidant assay (CAA), to evaluate the antioxidant activity of isorhamnetin 3-*o*-robinobioside (I3- *O*-Rob) and its original extract, ethyl acetate extract (EA extract) from *N. retusa* leaves which would serve as a more suitable method to measure.

## Methods

### Chemicals

All the organic solvents were obtained from Carlo ERBA (Paris, France). L-glutamine was purchased from GIBCO BRL Life technologies (Grand Island, NY, USA). The *N*-(1-naphtyl) ethlenediaminedihydrochloride (EDTA) was purchased from Sigma–Aldrich (Steinheim, Germany). RPMI-1640, foetal bovine serum and gentamicin were bought from GIBCO BRL Life technologies (Grand Island, NY, USA) Folin–Ciocalteu reagent and 2,2′-azobis (2-amidinopropane) dihydrochloride (ABAP) were purchased from Wako Chemicals USA, Inc. (Richmond, VA). 2′,7′-dichlorofluorescin diacetate (DCFH-DA) was purchased from Sigma-Aldrich, Inc. (St. Louis, MO). Dimethyl sulfoxide and acetic acid were obtained from Fisher Scientific (Pittsburgh, PA). Sodium carbonate, acetone, and methanol were obtained from Mallinckrodt Baker, Inc. (Phillipsburg, NJ).

### Plant material

*Nitraria retusa* was collected in December 2006 from the saline soils at Sahline, a region in the center of Tunisia. Identification was carried out by Pr. M. Cheieb (Department of Botany, Faculty of Sciences, University of Sfax, Tunisia) according to the Flora of Tunisia [11,12]. A voucher specimen (N.r-12.06) was kept at our laboratory for future reference. The leaves were shade-dried, powdered and stored in a tightly-closed container for further use.

### Preparation of ethyl acetate extracts from *Nitraria retusa* leaves

Three hundred and fifty grams of powder, from dried leaves, were sequentially extracted in a Soxhlet apparatus (6 h) (AM Glassware, Aberdeen, Scotland, United Kingdom) with hexane, chloroform, ethyl acetate and methanol solvents. We obtained the corresponding extract for each solvent. They were concentrated to dryness and kept at 4°C.

### Fractionation methods and structural identification of the purified compound

The ethyl acetate extract was fractionated by vacuum liquid chromatography (VLC) on a silica gel column and rechromatographed over RP18 column using medium liquid pression column (MLPC). Four sub-fractions were gathered, their purity was verified by thin layer chromatography, then identified by comparison of their Nuclear Magnetic Resonance (NMR) data to the literature to enable the identification of compound **1**.

### Cell culture and assay for cytotoxic activity

Human chronic myelogenous leukaemia CML cell line K562 was obtained from the American Type Culture Collection (Rockville, MD, USA). Cells were cultured in RPMI-1640 medium supplemented with 10% (v/v) foetal calf serum, 0.1 mg/ml gentamicin and 2 mM L-glutamine as complete growth medium, and were incubated at 37°C in an incubator with 5% CO_2_, in humidified atmosphere. Every 2 days, cells were split for subculture with fresh medium.

The cytotoxicity of *N. retusa* extract and I3- *O*-Rob against the K562 cells were estimated by the 3-(4,5-dimethylthiazol-2-yl)-2,5-diphenyltetrazolium bromide (MTT) assay, previousely described by Boubaker et al., [10].

Evaluation of the antioxidant capacity of EA extract and I3-*O*-Rob against lipid peroxidation provoked by H_2_O_2_, using the thiobarbituric acid reactive substance (TBARS) assay:

The method known as thiobarbituric acid reactive species (TBARS) assay concerns the spectrophotometric measurement of the pink pigment produced through the reaction of thiobarbituric acid (TBA) with malondialdehyde (MDA) and other secondary lipid peroxidation products. TBARS were determined according to the assay described by Ohkawa [13]. The cells (3.5 x 10^7^ cells/ml) were exposed to various concentrations of each sample (250, 500 and 1000 μg/ml of I3-*O*-Rob and 200, 400 and 800 μg/ml of EA extract) in the incubation medium for 2 h, followed by incubation with 50 μM H_2_O_2_ for 2 h. The doses ranges of the different tested compounds were chosen on basis of their cytotoxic activity. The cells were washed with PBS, pelleted and homogenized in 1.15% KCl. The samples were combined with 0.2 ml of 8.1% SDS, 1.5 ml of 20% acetic acid and 1.5 ml of 0.8% thiobarbituric acid. The mixture was brought to a final volume of 4 ml with distilled water and heated to 95°C for 120 min. After cooling for 10 min on ice, 5.0 ml of a mixture of n-butanol and pyridine (15:1 v/v) were added to each sample, and the mixture was shaken vigorously. After centrifugation at 825 g for 10 min, the supernatant fraction was isolated and the absorbance was measured at 532 nm. The lipid peroxidation effect was expressed as equivalent of MDA. Data were reported as mean ± SD for triplicate determinations.

### Cellular antioxidant activity (CAA) assay

Human myelogenous erythroleukaemia K562 cells were seeded at a density of 6 × 104/well on a 96-well microplate. Twenty-four hours after seeding, the growth medium was removed and the wells were washed with PBS. Triplicate wells were treated for 1 h with 100 μL of extracts plus 25 μM DCFH-DA dissolved in treatment medium. The wells were washed with 100 μL of PBS, then 600 μM ABAP solution was applied to the cells in 100 μL of PBS, and the 96-well microplate was placed into a Fluoroskan Ascent FL plate-reader (ThermoLabsystems, Franklin, MA) at 37°C. Emission at 538 nm was measured with excitation at 485 nm, every 5 min for 1 h. Each plate included triplicate control and blank wells: The control wells contained cells treated with DCFH-DA and oxidant ABAP; the blank wells contained cells treated with PBS without oxidant [14-16].

### Quantification of CAA

After blank subtraction from the fluorescence readings, the area under the curve of fluorescence versus time was integrated to calculate the CAA value at each concentration of the samples as follows:

(1) $$\text{CAA unit}=1-\left(\int \text{SA}\;/\int \text{CA}\right)\times 100$$

where ∫SA is the integrated area under the sample fluorescence versus time curve and ∫CA is the integrated area from the control curve. The median effective dose (IC_50_) was determined for the pure phytochemical compounds and the leaf extract from the median effect plot of log (*f*a/ *f*u) versus log (dose), where *f*a is the fraction affected and *f*u is the fraction unaffected by the treatment. To quantify the intraexperimental variation, the IC_50_ values were stated as mean SD for triplicate sets of data obtained from the same experiment [14-16].

### Comet assay

The Comet assay with human lymphocytes was used to detect DNA damage. Before each experiment, frosted microscope slides were precoated with 2 layers (100 μl) of normal agarose (1% in milli-Q water) and left at room temperature to allow agarose to dry. The cells were treated during 24 h with different concentrations of the tested samples. The treated cells were stressed with 75 μM H_2_O_2_ for 2 h. The cell dilution (5 × 10^5^ cells in 60 μl) was mixed with an equal volume of low-melting point agarose (1.2% in PBS). This agarose cell suspension (120 μl) was spread onto each precoated slide and covered with a cover slip. After 10 min on ice, the cover slip was gently removed, and the slides were placed in a tank filled with the lysate buffer (2.5 M NaCl, 100 mM EDTA, 10 mM Tris–HCl, 1% sodium sarcosinate pH 10, 1% of Triton X-100, and 10% DMSO). They were immersed for 1 h in this buffer (4°C, in the dark). The slides were then transferred into the electrophoresis buffer (NaOH 10 N, EDTA 200 mmol/l, pH 13 in deionized water) for 20 min at room temperature in darkness. Electrophoresis was carried out for 15 min at 25 V, 300 mA. Finally, the slides were gently rinsed with neutralization solution (0.4 M Tris–HCl, pH 7.5) 3 times for 5 min each. Staining of DNA was accomplished using 50 μl of ethidium bromide solution (20 μg/ml in PBS) per slide [17]. The slides were examined using an epifluorescence microscope (Zeiss Axioskop 20; Carl Zeiss, Microscope Division, Oberkochen, Germany).

### Quantification of the comet assay

A total of 100 comets on each scored slide for each sample concentration were visually-scored according to the relative intensity of fluorescence in the tail and classified as belonging to one of five classes. We utilised three slides for each extract concentration, and the experiments were repeated three times. Each comet class was given a value of 0, 1, 2, 3, or 4 (from undamaged, 0 to maximally damaged, 4) as described previously by Collins et al. [18]. The total score of DNA damage was calculated by the following equation:

(2) $$\begin{array}{l} \;\text{Total DNA damage}\left(\text{TDD}\right)=(\text{percentage of cells in class}\;0\times 0)+)\text{percentage of cells in class}\;1\times 1)+(\text{percentage of cells in class}\;2\times 2)+(\text{percentage of cells in class}\;3\times 3)+(\text{percentage of cells in class}\;4\times 4)\\ \text{Consequently},\;\text{the total score was ranging from}\;0\;\text{to}\;400. \end{array}$$

The inhibition percentage of Tail DNA (%) was calculated relative to DNA damage in the control group cells treated with H_2_O_2_ only by the following formula:

(3) $$\text{The}\;\text{inhibition}\;\text{percentage}\;\text{of}\;\text{Tail}\;\text{DNA\%}=\left[1-\left(\text{TDD}\;\text{of}\;\text{treated}\;\text{cells}\;\text{with}\;\text{extract}\;\text{and}\;{\text{H}}_{2}{\text{O}}_{2}-\text{TDD}\;\text{of}\;\text{untreated}\;\text{cells}\right)/\left(\text{TDD}\;\text{of}\;\text{stressed}\;\text{cells}\;\text{only}\;\text{by}\;{\text{H}}_{2}{\text{O}}_{2}-\text{TDD}\;\text{of}\;\text{untreated}\;\text{cells}\right)\right]x100\text{.}$$

### Statistical analysis

Data were collected and expressed as the mean ± standard deviation of three independent experiments and analysed for statistical significance from positive control. The data were tested for statistical differences by student test, differences were considered to be significant when *p* < 0.05.

## Results

### Phytochemical study of *Nitraria retusa* ethyl acetate leaf extract (EA) and isolation and structural identification of the purified compound

**Figure 1:**
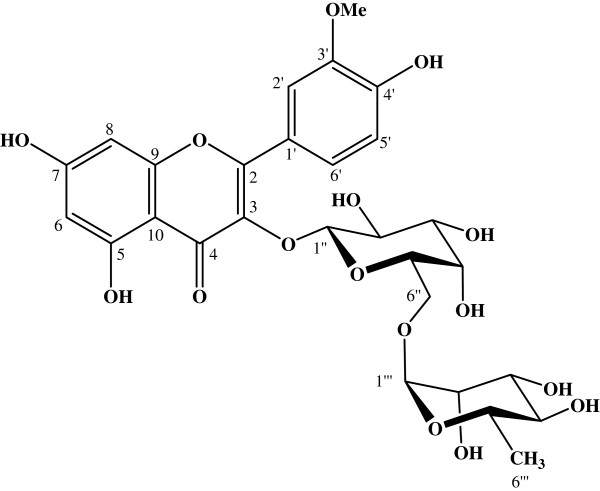
**Isorhamnetin 3-*O*-robinobioside structure (I3-*O*-Rob).**

Using 350 g of powder from leaves of *N. retusa*, we obtained 2.8 g of EA extract, corresponding to yield of 0.8%. The phytochemical study of *N. retusa* EA extract showed the presence of high quantities of flavonoids (193.33 μg/ml of quercetin equivalents). The structure of compound **1,** purified from EA extract, was established and the compound was identified as a flavonol, isorhamnetin3- *o*-robinobioside (I3- *O*-Rob) (Figure 1).

### Cytotoxic activity

The Cytotoxicity study showed that the tested concentrations of I3-*O*-Rob strongly inhibited the malignant tested cell population growth (IC_50_ values was 500 μg/ml). However, after incubation with 200, 400 and 800 μg/ml of EA extract, the inhibition percentages of the cell population growth were 25.74%, 31.39% and 36.78% respectively.

### Effect of *N. Retusa* products on lipid peroxidation induced by H_2_O_2_

The reaction of MDA with TBA has been widely adopted as a sensitive assay method for lipid peroxidation. The effect of the different concentrations of *Nitraria retusa* products on malondialdehyde (MDA) production induced by H_2_O_2_ in K562 cells is shown in (Table 1). I3-*O*-Rob showed a protective effect against lipid peroxidation induced by H_2_O_2_ at all the tested concentrations. The lipid peroxidation effect was evaluated as MDA equivalent produced in the presence of H_2_O_2_ (50 μM) and 1000, 500, 250 μg/ml of I3-*O*-Rob. These values were (respectively 100, 150, and 180 nM) lower than the one obtained with H_2_O_2_ alone (225 nM). However, EA extract did not exhibit any protective effect against H_2_O_2_ induced lipid peroxidation.

**Table 1:** **Lipid peroxidation inhibitory activity, in K562 cells treated with isorhamnetin 3-*o*-robinobioside (I3-*O*-Rob) and ethyl acetate (EA) extract, against H**_**2**_**O**_**2**_**(50 μM) induced peroxidation**

**Extracts**	**Concentration (μg/ml)**^**a**^	**Concentration of MDA (nM)**
**Isorhamnetin 3-*O*-robinobioside**	250	180 ± 11 *
	500	150 ± 10 *
	1000	100 ± 9 *
**Ethyl acetate extract**	200	240 ± 5
	400	235 ± 10
	860	220 ± 10
**H**_**2**_**O**_**2**_	50 μM	225 ± 5

^a^ results are represented by the means ± SD of three experiments.

(*) *p* < 0.05 means a significant difference between the treated cells with isorhamnetin 3- *o*-robinobioside (I3- *O*-Rob) and ethyl acetate (EA) extract, and cells treated with hydrogen peroxide (H_2_O_2_).

### Cellular antioxidant activity (CAA) assay

**Figure 2:**
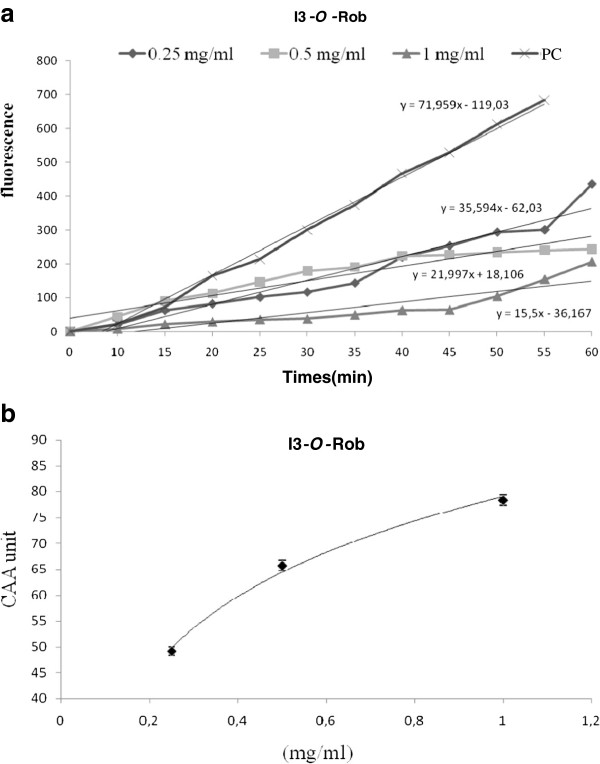
**a. Cellular Antioxidant Activity (CAA) of isorhamnetin-3-*o*- robinobioside (I3-*O*-Rob) K562 treated cells + 2 ′, 7′-dichlorofluorescin (DCFH) + 2,2′-azobis (2-amidinopropane) dihydrochloride (ABAP). PC: Untreated cells + 2 ′, 7′-dichlorofluorescin (DCFH) + 2,2′-azobis (2-amidinopropane) dihydrochloride (ABAP). b**: Dose–response curve for the inhibition of oxidation of the free-radical 2 ′, 7′-dichlorofluorescin (DCFH) to DCF in K562 cells using the cellular antioxidant activity assay in the presence of different concentration of isorhamnetin-3- *o*-robinobioside (I3- *O*-Rob) of *Nitraria retusa* (mean ± SD, n = 3).

**Figure 3:**
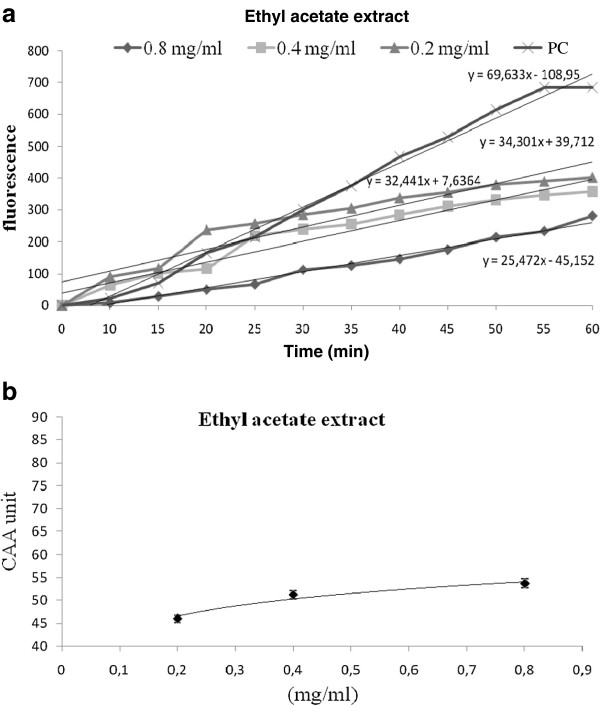
**a. Cellular Antioxidant Activity (CAA) of ethyl acetate extract (EA extract) K562 treated cells + 2 ′, 7′-dichlorofluorescin (DCFH) + 2,2′-azobis (2-amidinopropane) dihydrochloride (ABAP). PC: Untreated cells + 2 ′, 7′-dichlorofluorescin (DCFH) + 2,2′-azobis (2-amidinopropane) dihydrochloride (ABAP). b**. Dose–response curve for the inhibition of oxidation of the free-radical 2 ′, 7′-dichlorofluorescin (DCFH) to DCF in K562 cells using the cellular antioxidant activity assay in the presence of different concentrations of ethyl acetate extract (EA extract) (mean ± SD, n = 3).

In order to follow the antioxidant effect of EA extract and I3-*O*-Rob, at the intracellular environment, we used the test of Cellular Antioxidant Activity. The products obtained from the leaves of *N. retusa* showed a significant cellular antioxidant activity. I3- *O*-Rob and ethyl acetate extract have, respectively IC_50_ values of 0.225 mg/ml (Figure 2) and 0.31 mg/ml (Figure 3) in K562 cells.

### Comet assay

The induction of DNA damage in human leukemia K562 cells after exposition to different *Nitraria retusa* products was investigated using the Comet assay. Firstly, we studied the direct effect of EA extract and I3- *O*-Rob on K562 cells DNA. Data are reported as Total DNA damage (TDD) in Table 2. It was revealed that both tested samples induced no genotoxicity as no significant difference was detected between the TDD of the EA extract (TDD values were 253, 261 and 257 at concentrations of (200, 400 and 800 μg/ml) respectively and I3-*O*-Rob (TDD values were 260, 255 and 254 at concentrations of (250, 500 and 1000 μg/ml) respectively in one hand, and the negative control (non-treated cells; TDD = 252 ± 6) in the other hand. On the opposite, a significant increase of the Total DNA damage was observed in K562 cells exposed to 75 μM of H_2_O_2_ (TDD = 360 ± 5) compared to the untreated cells. The alkaline single-cell gel electrophoresis (comet) assay was also performed in order to elucidate antigenotoxicity effect shown by the different *Nitraria retusa* products against H_2_O_2_-induced DNA damage in K562 cells. I3-*O*-Rob and EA extract were effective in reducing the genotoxicity induced by 75 μM of H_2_O_2_. Indeed, I3-*O*-Rob and EA extract decreased the tail extent of comets in a dose-dependent manner, by 80.55% and 77.77% respectively at the highest tested concentrations (1000 and 800 μg/ml). These products inhibited genotoxicity induced by hydroxyl radicals.

**Table 2:** **Treatment of K562 cell DNA with Isorhamnetin 3-*O*-robinobioside (I3-*O*- Rob) and ethyl acetate extract (EA extract) in the presence and abscence of H**_**2**_**O**_**2**_**in comet test**

**Comet assay on genomic DNA of K562 cells treated with Isorhamnetin 3-*O*-robinobioside (I3-*O*- Rob) and ethyl acetate extract (EA extract)**			**Inhibitory effect of Isorhamnetin 3-*O*-robinobioside (I3-*O*- Rob) and ethyl acetate extract (EA extract) on the genotoxicity of K562 cells against H**_**2**_**O**_**2**_	
Extracts	Concentrations μg/ml)	Total DNA Damage (TDD)	Total DNA Damage (TDD)	Inhibition percentage (%)
T	-	252 ± 6	252 ± 6	
H_2_O_2_	75 μM	360 ± 5	360 ± 5	
Ethyl Acetate extract	200	253 ± 5	298 ± 4	57.40*
	400	261 ± 4	287 ± 5	67.6*
	800	257 ± 6	276 ± 4	77.77*
Isorhamnetin 3-*O*-robinobioside	250	260 ± 5	297 ± 6	58.33*
	500	255 ± 4	286 ± 5	68.51*
	1000	254 ± 4	273 ± 5	80.55*

* P < 0.05 compared to negative control without Isorhamnetin 3-*O*-robinobioside (I3- *O*- Rob) and ethyl acetate extract (EA extract) by student test. (results are represented by the means ± SD of three experiments).

## Discussion

Some studies have shown that flavonoids induce apoptosis of various tumour cells including K562cells. This effect has also been observed in other tumor cell lines from gastric, colon and lung carcinomas [19]. In addition, flavonoids inhibited tumor growth through cell cycle arrest and induced apoptosis through a p53-dependent mechanism [20].

Flavonoids may intervene at the different levels of lipid peroxidation process [21]. They are able to directly capture radical species and thus interrupt the step of propagating radical [22]. Moreover, being good chelating, they are capable to coordinate the free iron. Finally, flavonoids on the surface of the membranes are able to regenerate vitamin E, an essential antioxidant in the cell membranes protection [23].

On the other hand, flavonoids are powerful antioxidants against free radicals because they act as “radical-scavengers”, the antioxidant capacity of a flavonoid is linked to its particular chemical structure. We believe that the presence of the double bond C2–C3 in conjugation with a 4-oxo in the I3-*O*-Rob structure participates in the antioxidant potenty of the purified compound (I3- *O*-Rob), as well as its original extract (EA extract). This hypothesis is in accordance with the results described by Rice-evans et al. [24] and Van Acker et al. [25], who reported that the antioxidant activity of quercetin (flavonoids with C2-C3 and C4 carbonyl function on cycle C) is greater than dihydroquercetin (flavonoids without double band C2-C3 but with C4 carbonyl function on cycle C). Rice-Evans et al. [24] explained this property by the conjugation of rings A and B that allows the resonance of the aromatic ring, thus stabilizing the phenoxy radical.

The presence of 5-OH and 7-OH functions, together with carbonyl group may be involved in I3-*O*-Rob antioxidant potenty as it has been advanced by Edenharder and Grünhage [26], who reported that the hydroxylation of C5 and C7, together with carbonyl group in C4 meets the criteria of potent radical scavenger.

The antioxidant potenty of I3-*O*-Rob can also be related to the presence of the OH group at C4 of ring B of the tested flavonoid. This hypothesis is supported by the studies of Mathiesen et al. [27] that showed that C4 hydroxylated angolétine are more antioxidant than C4 O-methylated myrigalone.

Likewise, we believe that the presence of sugar moiety participates in antioxidant activity of I3-*O*-Rob. This observation is in accordance with the results reported by Hayder et al. [28] stipulating that glycosylated myricetin improves the antioxidant capacity of the corresponding aglycone.

EA extract exhibited a lower antioxidant capacity compared to its major constituent, I3-*O*-Rob. This could be explained by the presence, in the extract, of compounds with antagonistic effects and also that I3- *O*-Rob was diluted in the original EA extract.

Obviously, flavonoid antioxidant capacity is also linked to another structural element, which is the presence of methoxyl group on the ring B of I3-*O*-Rob that could participate in the antioxidant potential of this molecule. Kawaii et al. [29] reported that many flavonoids with methoxyl substitutions are considered potent anticancer flavonoids.

When treated with H_2_O_2_, the predominant lesion in cell DNA is the strand breaks and base oxidation. Such damages can increase the risk of cancer development [30]. The protective action of the tested compounds can be explained by their ability to penetrate through the cell membrane and to interrupt the radical chain induced by H_2_O_2_, thus, allowing the prevention or reduction of free radical formation, which are responsible for cellular macromolecules damage.

Many studies reported the capacity of natural antioxidants in influencing disease progression. This property is closely related to their ability to reduce DNA damage, mutagenesis, carcinogenesis and inhibition of pathogenic bacterial growth [31].

Indeed, flavonoids have been shown to be effective scavengers of reactive oxygen species (ROS), and it has been suggested that flavonoid anticancer activities greatly depend on their antioxidant and chelating properties [32,33].

We deduce that the antigenotoxic activity can be ascribed not only to the antioxidant effect of these molecules but also to other additional mechanisms like DNA repair enzyme induction as it was evidenced by Hayder et al. [28] who reported that, compared to myricetin alone, the antioxidant enzymes and DNA repair enzyme expression, are modulated in the presence of glycosylated myricetin, using a microarray system. In fact, Kooststra [34] demonstrated that flavonoids should neutralize the free radicals that promote mutations, when generated near DNA. Flavonoids can also protect the DNA by directly interacting with the mutagen agents, as in the induced chromosomal aberration by bleomycin alluded by Heo et al. [35]. Nevertheless, the inhibition of mutagenesis is often complex, acting through multiple mechanisms. Edenharder et al. [36] demonstrated a dual role of flavonoids as far as they do not only inhibit membrane-bound cytochrome P-450-dependent monooxygenases, but also inhibit various soluble enzymatic factors, suggesting interactions with biological membranes and effects on expression and fixation of DNA damages.

## Conclusions

The experiments described above demonstrated that isorhamnetin 3-*o*-robinobioside from *Nitraria retusa* exhibited significant antioxidant and antigenotoxic effects in human cells line. This work paves the way for studying this medicinal plant in an alternative phytotherapy alternative for a number of human degenerative disorders.

## Competing interests

The authors have declared no conflict and financial competing of interest.

## Authors’ contributions

BJ: Was responsible for the conception and design, testing and data acquisition, analysis and data interpretation and drafted the manuscript. BSM: made contribution to conception of the design of the antioxidant tests. SI: made contribution to conception of the phytochemical study. GK: made substantial contribution to conception and revised it critically for important intellectual content. CGL: made substantial contribution to conception and revised it critically for important intellectual content. All authors read and approved the final manuscript.

## Pre-publication history

The pre-publication history for this paper can be accessed here:

http://www.biomedcentral.com/1472-6882/12/135/prepub
